# A Broadband Millimeter-Wave Circularly Polarized Folded Reflectarray Antenna Based on Transmissive Linear-to-Circular Polarization Converter

**DOI:** 10.3390/mi16060711

**Published:** 2025-06-14

**Authors:** Yue Cao, Zhuwei Wang, Qing Wang, Mingzhu Du, Miaojuan Zhang

**Affiliations:** School of Information Science and Technology, Nantong University, Nantong 226019, Chinawq2430310033@163.com (Q.W.); mzdu@ntu.edu.cn (M.D.);

**Keywords:** folded reflectarray antenna (FRA), metasurface, transmissive linear-to-circular polarization converter, millimeter-wave (mm-W), wideband, circularly polarized (CP) antennas

## Abstract

In this paper, a wideband circularly polarized folded reflectarray antenna (CPFRA) based on a transmissive linear-to-circular polarization converter is proposed. The CPFRA consists of a primary reflector and a sub-reflector. To achieve broadband performance, a metasurface-based RA element on the primary reflector surface and a transmissive linear-to-circular polarization converter on the sub-reflector surface are applied. Moreover, the transmissive linear-to-circular polarization converter on the sub-reflector surface helps convert linear polarization to circular polarization. To verify the proposed CPFRA, a prototype is designed, fabricated, and tested. The measured results exhibit that the proposed CPFRA presents a 3 dB gain bandwidth of 27.4% and a 3 dB axial ratio bandwidth of 23%. The CPFRA achieves a peak gain of 21.2 dBi with an aperture efficiency of 27.2%. The proposed CPFRA is a promising candidate for millimeter-wave (mm-W) satellite communication applications because of its advantages of high gain, low cost, low profile, and broad bandwidth.

## 1. Introduction

With the rapid development of the new generation of mobile communication, radar systems, satellite communication, and other wireless communication systems [1], antennas with a high gain, wide bandwidth, and low profile have become research trends for modern millimeter-wave (mm-W) antennas. The reflectarray antenna (RA), as a type of antenna that combines the characteristics of a planar microstrip array antenna and a high-gain reflector antenna, has the advantages of a high gain, simple feeding, easy processing, and a low cost [2]. Moreover, due to the fact that the RA adopts the spatial feeding method, the profile between the feed source and the array surface of the RA is relatively high, making it bulky and difficult to integrate with other systems [3]. Compared with the RA, a folded reflectarray antenna (FRA) can reduce the profile between the feed source radiator and the aperture of the reflectarray surface effectively by using dual-polarized elements for polarization rotation and a polarizer grid right above them [4,5]. Many publications have reported FRAs with various performances, such as multibeam [6], full-metal structure [7], dual-frequency [8,9], ultra-low-profile [10] and planar feed source radiator arrays [11].

Among FRAs, circularly polarized folded reflectarray antennas (CPFRAs) are more appropriate for long-distance communication systems, such as satellite communication systems and radio communication systems, because of the reduction in polarization loss and suppression of multipath fading [12]. Recently, many research works related to CPFRAs have been published. According to the polarization mode of the feed source antenna, CPFRAs can be classified into two types. The first type of CPFRA adopts a circularly polarized antenna as the feed source radiator [13,14]. In [13], a folded reflectarray antenna using a CP conical horn antenna as the feed source was proposed. The physical fabrication of the CP conical horn antenna is complex and expensive. In [14], a folded reflectarray antenna using a planar CP microstrip patch array antenna as the feed source was proposed. Planar feeding can reduce the profile height of the antenna and is easy to integrate with other components, but the design of the feed source is often complex. The second type of CPFRA uses a linearly polarized (LP) antenna as the feed source radiator [15,16,17]. In [16], a folded reflectarray antenna based on an LP horn antenna was proposed. The design of the LP horn feed is simple, but it is accompanied by the disadvantages of a relatively high profile and high production costs. In [17], a folded reflectarray antenna using an open waveguide antenna as the feed source was proposed. The design of the waveguide antenna is simple and easy to process. In addition, a linear-to-circular polarization converter is needed as the sub-reflector in the second CPFRA so that the final electromagnetic waves radiated out are circularly polarized. Therefore, when taking the design simplicity and cost-effectiveness into consideration, the second type of CPFRA, which uses a linearly polarized feed source radiator, is preferred.

In this work, a CPFRA with a low profile and broad bandwidth based on a transmissive linear-to-circular polarization converter for millimeter-wave applications is studied. This CPFRA is composed of a linear horn antenna as the feed radiator, a metasurface-based element as the basic structure of the primary reflector surface, and a transmissive polarization converter as the sub-reflector surface. This manuscript is organized as follows: Firstly, Section Ⅱ introduces the architecture of the CPFRA. The primary reflector surface has the functions of polarization twisting and phase compensation. It can convert the linearly polarized electromagnetic waves (EWs) emitted by the feed source into linearly polarized EWs with vertical polarization and perform phase compensation. The sub-reflector surface reflects the incident linearly polarized electromagnetic waves from the feed radiator for the first time. When the reflected electromagnetic waves are rotated by 90° and incident on the sub-reflector surface again, they are converted into circular polarization and transmitted out. Then, the structures and performances of the elements of the primary reflector surface and the sub-reflector surface are displayed and analyzed, respectively. To verify the proposed CPFRA, a prototype with 144 elements is designed, fabricated, and tested in Section Ⅲ. Finally, Section Ⅳ concludes the design.

## 2. The Principle and Element Design of the CPFRA

### 2.1. The Principle of the Proposed CPFRA

**Figure 1:**
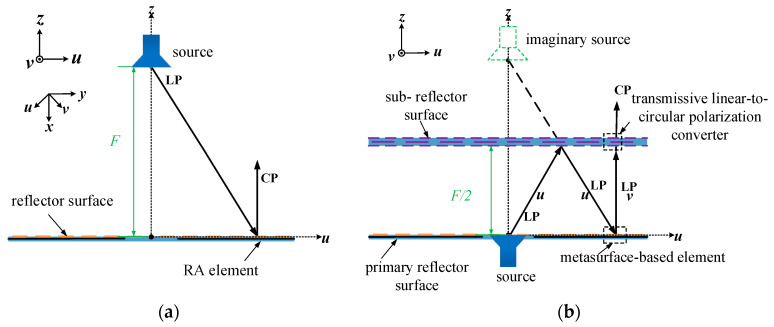
Architectures of (**a**) the traditional circularly polarized RA and (**b**) the proposed circularly polarized FRA.

Figure 1 shows the architecture of the proposed CPFRA, which is composed of three parts: a linearly polarized feed radiator, a primary reflector surface, and a sub-reflector surface. Here, the *u*-direction indicates the direction within the *xoy* plane that forms a −45° angle with the positive direction of the *x*-axis, and the *v*-direction indicates the direction within the *xoy* plane that forms a 45° angle with the positive direction of the *x*-axis.

**Figure 2:**
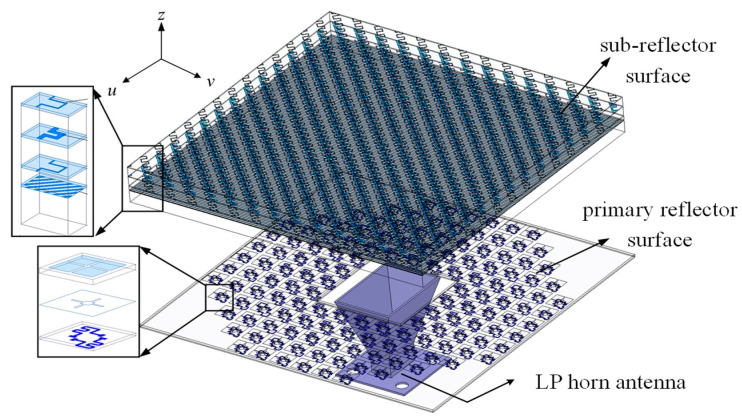
Three-dimensional structure and exploded view of the proposed antenna.

The working principle of this antenna is as follows: When the EWs along the *u*-polarization direction from the feed radiator are incident on the bottom of the sub-reflector surface, they are reflected back and incident on the primary reflector surface. The elements on the primary reflector surface receive the EWs polarized along the *u*-polarization direction and perform phase compensation and polarization rotation by 90°. Then, the modulated EWs polarized along the *v*-polarization direction are incident on the primary reflector surface again. At this time, the element based on the transmissive linear-to-circular polarization converter on the sub-reflector surface can pass through the EWs polarized along the *v*-polarization direction and convert them into CP EWs for radiation. To summarize, the requirements for each part of the antenna are as follows in this architecture: For the primary reflector surface, the functions of phase compensation and polarization rotation are required. For the sub-reflector surface, the functions of reflecting EWs polarized in the *u*-axis direction, transmitting EWs polarized in the *v*-axis direction, and converting EWs polarized in the *v*-axis direction into circularly polarized EWs are required. A 3D diagram of the final design for the folded reflectarray antenna is shown in Figure 2.

### 2.2. Geometry and Performance of the Metasurface-Based Element on the Primary Reflector Surface

#### 2.2.1. Geometry of the Metasurface-Based Element

**Figure 3:**
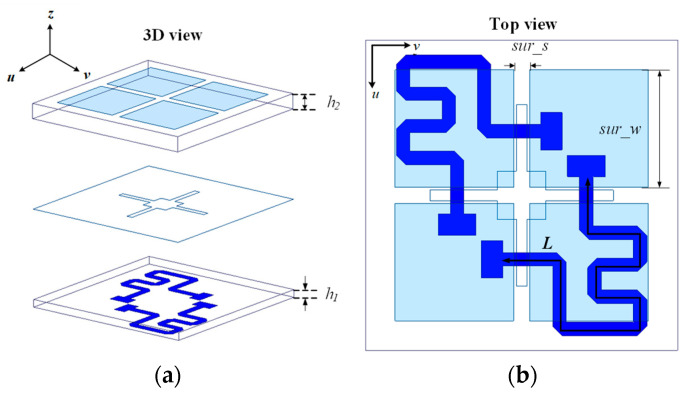
(**a**) Three-dimensional view; (**b**) top view.

In order to realize the architecture proposed in Section 2.1, a metasurface-based element on the primary reflector surface is designed. Figure 3a shows a 3D view of the proposed element. The metasurface-based element is composed of two dielectric layers and three metal layers. The top layer is a 2 × 2 square patch, the middle layer is a metal ground layer with a cross-shaped slot, and the bottom layer is a microstrip line layer. The dielectric substrate of the metasurface-based element is made of Rogers Duroid 4003 material (with *ε_r_* = 3.55 and tan*δ* = 0.0027 at 10 GHz). The thicknesses of the upper substrate and the lower substrate are 0.508 mm and 0.254 mm, respectively. Figure 3b shows the top view of the metasurface-based element. The length of the side of the element is 5.8 mm. The length of the square patch is *sur_w* = 2.2 mm. The distance between two adjacent patches is *sur_s* = 0.3 mm. Because of the introduction of the metasurface, broadband performance can be achieved. In addition, a reflective phase of more than 360° can be adjusted by changing the length of the microstrip line (L). The detailed working principle and design steps can be found in [18].

#### 2.2.2. Performance of the Metasurface-Based Element

**Figure 4:**
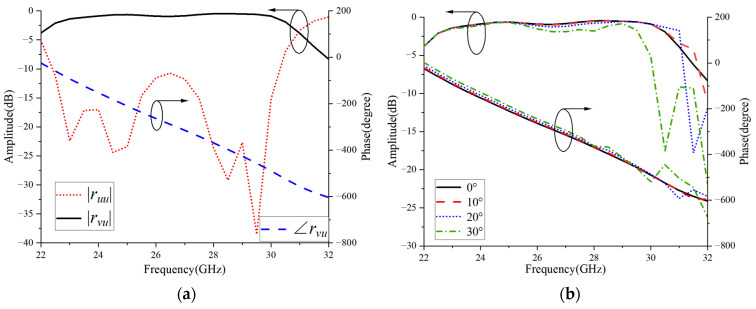
Reflection amplitude and phase responses based on metasurface elements under (**a**) normal incident and (**b**) oblique incident conditions.

The metasurface-based element on the primary reflector surface is simulated using the commercial simulation software HFSS19.0. As shown in Figure 4a, *r_uu_* indicates that the polarization direction of the incident wave is along the *u*-axis and the polarization direction of the reflected wave is also along the *u*-axis. *r_vu_* indicates that the polarization direction of the incident wave is along the *u*-axis and that of the reflected wave is along the *v*-axis. |*r_uu_*| and |*r_vu_*| represent the reflection amplitudes, and ∠*r_vu_* represents the reflection phase. The arrows in the curve point to the corresponding *y*-axis. Within the frequency band range of 22.4–30.1 GHz, |*r_uu_*| is always lower than −10 dB, indicating that the energy of the *u*-polarized wave reflected back by the metasurface-based element is very low. |*r_vu_*| is greater than −1.5 dB and close to 0 dB, indicating that the incident *u*-polarized wave is reflected as a *v*-polarized wave, suggesting that the metasurface-based element has excellent polarization torsional characteristics in the broadband range. Furthermore, from Figure 4a, we can also see that the ∠*r_vu_* phase curve changes almost linearly with frequency. This linear phase response is helpful for designing reflectarray antennas with wideband characteristics.

As shown in Figure 4b, the metasurface-based element still has good polarization conversion performance under oblique incidence. Within the frequency band range, the amplitude and phase changes are small and remain relatively stable.

**Figure 5:**
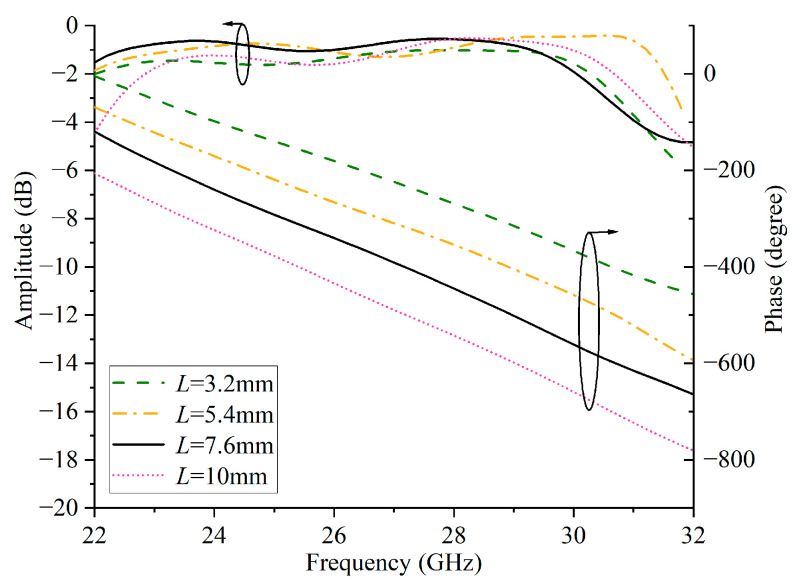
Simulated amplitude and phase of the metasurface-based element on the primary reflector surface.

The metasurface-based element achieves 360° reflection phase compensation by changing the length (L) of the microstrip lines. Figure 5 shows the result of phase compensation. When changing the length of the microstrip lines from 3.2 mm to 10 mm, the values of the reflective amplitude are more than −2 dB. Moreover, more than a 360° phase range can also be obtained. The simulation results show that the element has good performance.

**Figure 6:**
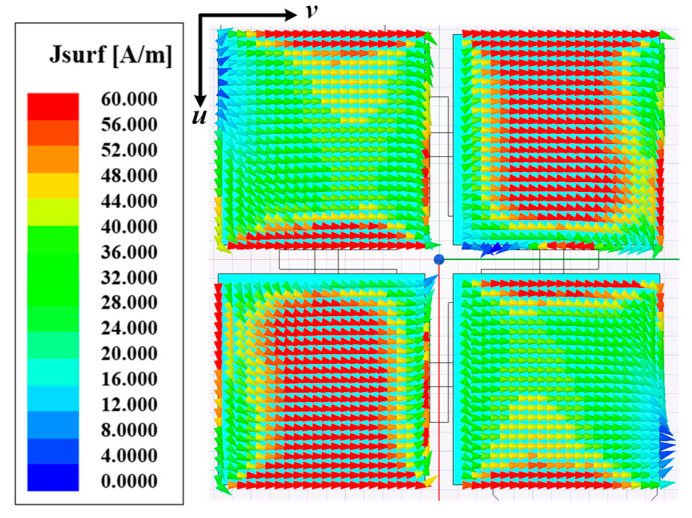
Surface current distribution of a 2 × 2 patch layer based on metasurface-based element.

Figure 6 shows the surface current distribution of a 2 × 2 patch layer based on a metasurface-based element. It can be seen from the figure that the 2 × 2 patch layer can be effectively excited to reflect and polarize EWs.

### 2.3. Design and Analysis of the Transmissive Linear-to-Circular Polarization Converter on the Sub-Reflector Surface

#### 2.3.1. Design of the Transmissive Linear-to-Circular Polarization Converter

**Figure 7:**
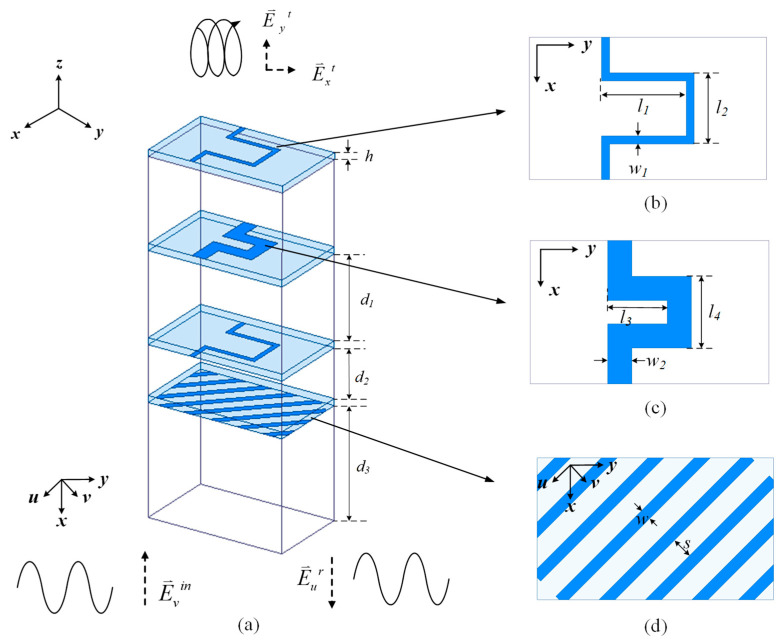
Geometry and performances of the transmissive linear-to-circular polarization converter on the sub-reflector surface: (**a**) 3D view, (**b**) top view, and (**c**) top view of the second layer of the mender line; (**d**) top view of the grid.

Based on the architecture proposed in Section 2.1, the sub-reflector surface needs to have the functions of converting LP to CP and polarization selection. Inspired by the linearly polarized grids and the transmission-type circularly polarized mender lines, a transmissive linear-to-circular polarization converter based on polarized grids and mender lines is proposed as the sub-reflector surface element. The geometry is shown in Figure 7. As shown in Figure 7a, ${{\overset{⃑}{E}}_{y}}^{t}$ represents the electric field of the transmitted EW along the *y*-polarization direction, ${{\overset{⃑}{E}}_{x}}^{t}$ represents the electric field of the transmitted EW along the *x*-polarization direction, ${{\overset{⃑}{E}}_{v}}^{in}$ represents the electric field of the incident EW in the *v*-polarization direction, and ${{\overset{⃑}{E}}_{u}}^{r}$ represents the electric field of the reflective EW in the *u*-polarization direction. The polarization converter is composed of a four-layer dielectric substrate, which is made of Rogers Duroid 4350 material with the same thickness *h*. Under each layer of dielectric substrate layer is an air layer. The thicknesses of the air layers are *d*_1_, *d*_2_, and *d*_3_. Each layer of the dielectric substrate is covered with metal on the bottom and without metal on the top. The top metal layer to the third metal layer are mender lines, and the top and third mender lines are the same. The top and second metal layers are displayed in Figure 7b,c. Figure 7d shows the bottom metal layer, which is composed of polarized grids. The detailed parameter values of the transmissive linear-to-circular polarization converter are listed in Table 1.

The working principle of the proposed transmissive linear-to-circular polarization converter is as follows: When the linearly polarized EWs along the *u*-polarization direction are incident to the transmissive linear-to-circular polarization converter on the sub-reflector surface, they are reflected back and cannot pass through this polarization converter because of the polarization selection characteristic of the polarized grids. However, the linearly polarized incident EWs along the *v*-polarization direction can pass through this structure and be converted into two orthogonal components with equal amplitudes and a phase difference of −90°. These two components can be synthesized to achieve a circular polarization characteristic.

#### 2.3.2. Analysis of the Transmissive Linear-to-Circular Polarization Converter

**Figure 8:**
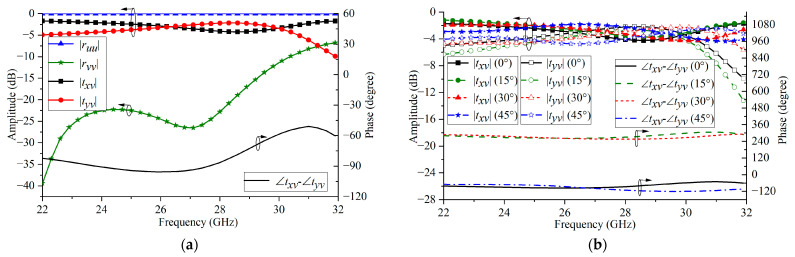
(**a**) Simulated amplitude and phase under vertical incidence and (**b**) under oblique incidence.

The simulated reflective and transmissive results are shown in Figure 8a. The symbol |*r_vv_*| represents the reflection coefficient of the linearly polarized incident wave along the *v*-polarization direction. |*t_yv_*| indicates the amplitude of the transmitted wave along the *y*-axis component when the incident wave is linearly polarized along the *v*-polarization direction. |*t_xv_*| represents the amplitude of the transmitted wave along the *x*-axis component when the incident wave is linearly polarized along the *v*-polarization direction. The simulation results show that when the incident wave is incident perpendicularly, the linearly polarized EW in the *x*-direction is reflected back and cannot pass through the polarization converter. The linearly polarized incident waves along the *y*-axis direction can pass through this polarization converter, and within the frequency band of 23–29 GHz, the amplitudes of the two orthogonal components of the transmitted waves are approximately equal, and the phase difference is −90° ± 15°. Figure 8b shows the transmission amplitude and phase difference responses of the linear-to-circular polarization converter under the oblique incidence condition. The results indicate that the proposed transmissive linear-to-circular polarization converter still has good polarization conversion performance under oblique incidence and is suitable for the design of broadband low-profile circularly polarized RAs.

## 3. Experimental Results and Analysis

### 3.1. Design of the LP Horn Antenna

**Figure 9:**
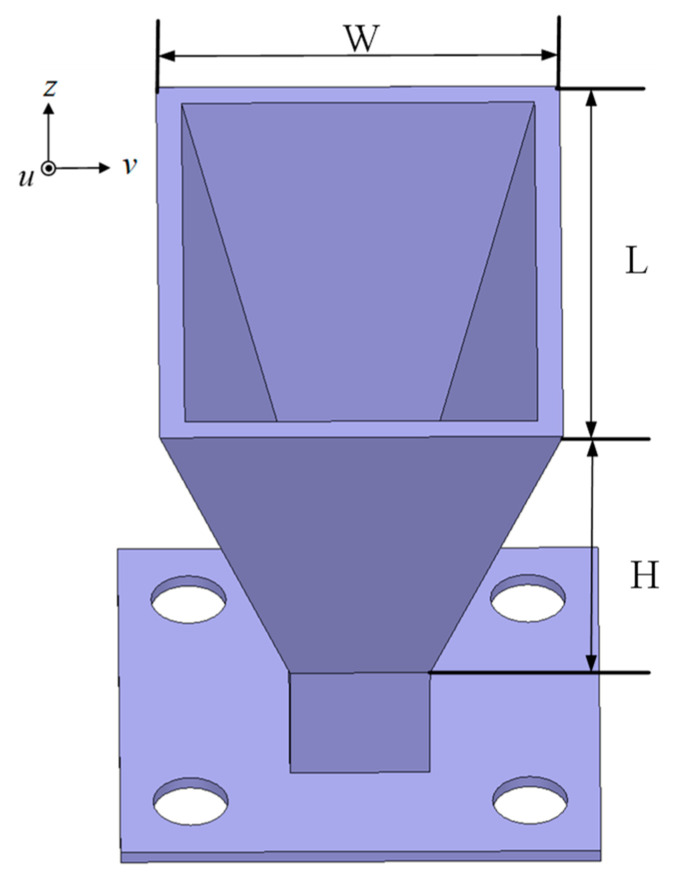
Three-dimensional view of the LP horn antenna.

In this design, the LP cone horn antenna as shown in Figure 9 is adopted as the feed source of the reflectarray antenna. The proposed LP horn antenna operates within the frequency band of 22–36 GHz. Therefore, a WR34 standard rectangular waveguide is connected to the cone speaker, and then a 2.92 mm coaxial adapter is used to convert the WR34 standard rectangular waveguide to achieve the connection between the horn antenna and the final test equipment. In order to facilitate physical testing, a flange plate is designed for the assembly of the test. Its dimensions are L = 20 mm, W = 14 mm, and H = 17 mm.

**Figure 10:**
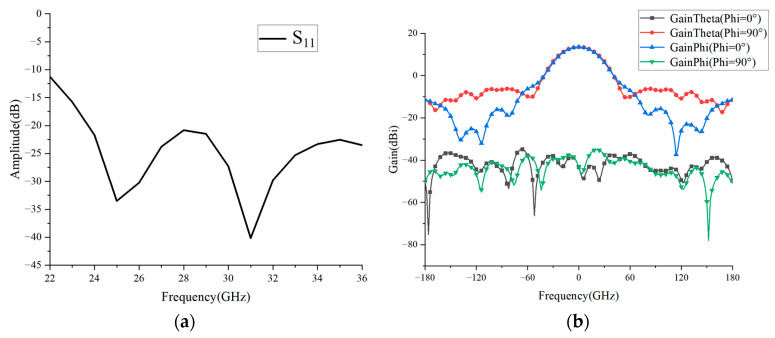
(**a**) Reflection coefficient and (**b**) normalized radiation pattern of the LP horn antenna.

Figure 10a shows the reflection coefficient of the LP horn antenna. It can be seen that within the frequency band range of 22–36 GHz, the reflection coefficient is always less than −10 dB. Figure 10b is the normalized radiation pattern of the speaker at a center frequency of 26 GHz. It can be seen that its peak gain is 13.5 dBi and the cross-polarization is less than −50 dB. If the −10 dB lobe width of this speaker is 64°, then the opposite half-angle is 32°.

### 3.2. Layout of the CPFRA

For verification, one broadband circularly polarized FRA prototype of 144 elements was designed based on the above architecture of the CPFRA for the satellite communication application in the millimeter-wave band. The FRA consists of 144 elements with an aperture area of 40.6 × 40.6*π* mm^2^ (12.3*πλ*_0_^2^). A linearly polarized pyramid horn with an aperture of 14 × 20 mm^2^ and a height of 17 mm is adopted as the feeding source, which is installed on the center. In order to take into account a better aperture efficiency, the distance between the primary reflector surface and the sub-reflector surface is 32.5 mm, which corresponds to a focal length (*F*) of 65 mm. The simulated −10 dB beam-width of the horn radiation pattern is 64°. Once the layout of the CPFRA is ready, the phase compensation of each element on the primary reflector surface can be determined accordingly.

### 3.3. Fabrication and Assembly of the CPFRA

**Figure 11:**
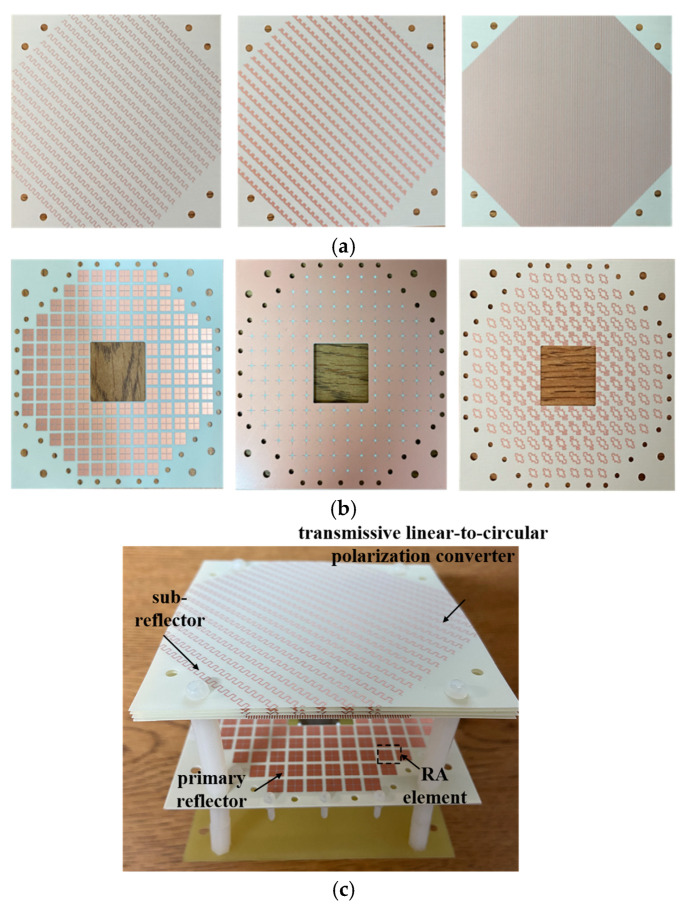
The photographs of the fabricated CPFRA. (**a**) Top view of the sub-reflector, (**b**) top view of the primary reflector, and (**c**) 3D view of the FRA.

The primary reflector surface and the sub-reflector surface were fabricated using the standard printed circuit board (PCB) process. The fabricated parts of the CPFRA are shown in Figure 11. Figure 11a is a photo of the transmissive linear-to-circular converter, which consists of the sub-reflector, and Figure 11b is a photo of the metasurface-based element, which consists of the primary reflector. The LP horn antenna was fabricated with metal materials. To assemble the FRA with the linearly polarized horn antenna, a substrate, several nylon struts, and screws were used, as shown in Figure 11c.

### 3.4. Experimental Results

**Figure 12:**
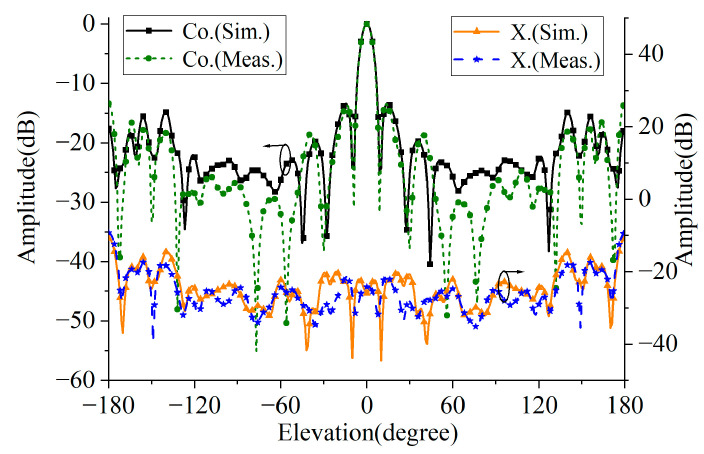
Simulated and measured normalized radiation patterns.

**Figure 13:**
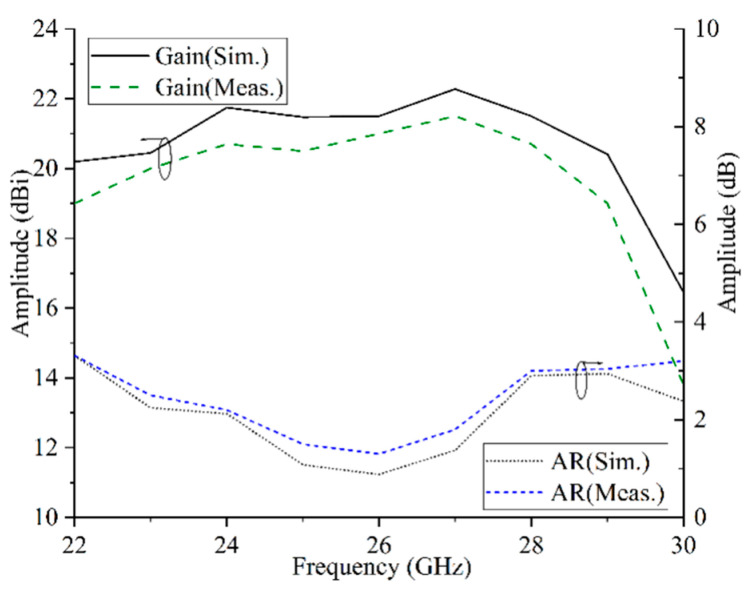
Measured and simulated gain and axial ratio curves versus frequency.

The proposed CPFRA is measured in a microwave anechoic chamber. The reflection characteristics of the antenna are measured by using a vector network analyzer (VNA). In addition, a coaxial-to-waveguide adaptor also needs to connect the test system and the feed horn. Figure 12 shows the measured normalized radiation patterns of the CPFRA at 26 GHz. It can be seen from the figure that the radiation pattern is consistent, the sidelobe is less than −13 dB, and the cross polarization is less than −15 dB. The curves of gain and aperture efficiency varying with frequency are shown in Figure 13. The difference between the simulation and test results mainly lies in the machining error and assembly errors. The FRA achieves a maximum gain of 21.2 dBi and an aperture efficiency of 27.2% at 26 GHz. In addition, the 3 dB gain bandwidth of the proposed FRA is 27.4% (22–29 GHz), and the 3 dB axial ratio (AR) bandwidth is 34%.

The performances of the proposed CPFRA together with other published works [15,19,20,21,22] are shown in Table 2. As can be seen from the table, compared with the non-folded circularly polarized reflectarray antenna [19,20,21,22], the profile of the proposed circularly polarized folded reflectarray antenna in this work is reduced to half of that without folding. In terms of performance, compared with [21], although the proposed antenna has a lower aperture efficiency, it can still achieve a higher 3 dB axial ratio (AR) bandwidth. Compared with the circularly polarized folded reflectarray antenna design with a linearly polarized source in [15], the circularly polarized folded reflectarray antenna based on the transmissive linear-to-circular polarization converter proposed in this work has an approximately equal aperture efficiency while having a wider 3 dB gain bandwidth and 3 dB AR bandwidth.

## 4. Conclusions

A broadband circularly polarized folded reflectarray antenna based on a transmissive linear-to-circular polarization converter is presented in this paper. The elements that make up the architecture of the CPFRA are designed and analyzed. The metasurface-based element on the primary reflector provides the broadband reflection and linear phase compensation. The transmissive linear-to-circular polarization converter on the sub-reflector provides the wideband polarization conversion. Compared with the conventional circularly polarized RA, the proposed FRA reduces the distance between the source and the primary reflector to half and achieves a 3 dB gain bandwidth of 27.4% (22.5–31.5 GHz) and a 3 dB AR bandwidth of 23%. The broadband low-profile circularly polarized reflectarray antenna proposed in this work has a low cost, low profile, and high bandwidth and can be applied well in millimeter-wave communication systems, especially in satellite communication systems.

## Figures and Tables

**Table 1:** Values of the polarization converter.

**Parameters**	***h***	***d*** **_1_**	***d*** **_2_**	***d*** **_3_**	***w*** **_1_**	***w*** **_2_**	***sur_w***
Value	0.508 mm	0.508 mm	1 mm	3.3 mm	2 mm	1.85 mm	2.2 mm
**Parameters**	***l*** **_1_**	***l*** **_2_**	***l*** **_3_**	***l*** **_4_**	***w***	***s***	***sur_s***
Value	0.3 mm	1.46 mm	0.2 mm	0.4 mm	0.2 mm	0.4 mm	0.3 mm

**Table 2:** Performance comparison with previously reported antennas.

Ref.	Frequency (GHz)	Polarization of the Source	Profile of RA	Aperture Efficiency (%)	Gain Bandwidth (%)	3 dB AR Bandwidth (%)
[19]	10	CP	F	59.94	30 (−1 dB)	30
[20]	15	CP	F	60	49.7 (−3 dB)	75
[21]	10	LP	F	39	17 (−1 dB)	11
[22]	10	LP	F	/	40 (−3 dB)	40
[15]	5.3	LP	F/2	27	9 (−3 dB)	5
This work	26	LP	F/2	27.2	27.4 (−1 dB)	23

## Data Availability

The original contributions presented in the study are included in the article, further inquiries can be directed to the corresponding author.
